# Lactate-induced metabolic signaling is the potential mechanism for reshaping the brain function - role of physical exercise

**DOI:** 10.3389/fendo.2025.1598419

**Published:** 2025-06-09

**Authors:** Xueqiang Zhu, Wenjia Chen, Ricardo A. Pinho, Anand Thirupathi

**Affiliations:** ^1^ School of Competitive Sports, Shandong Sport University, Rizhao, Shandong, China; ^2^ School of Physical Education, China University of Mining and Technology, Xuzhou, Jiangsu, China; ^3^ Graduate Program in Health Sciences, School of LifeSciences and Medicine, Pontifícia Universidade Católica doParaná, Curitiba, Brazil; ^4^ Faculty of Sports Science, Ningbo University, Ningbo, China

**Keywords:** lactate, physical exercise, metabolic signaling, energy homeostasis, brain health

## Abstract

Research into the effects of physical exercise on brain metabolism has revealed complex molecular mechanisms, with particular emphasis on lactate as a signaling molecule capable of transiently enhancing brain functions. This metabolite, once considered merely a byproduct of exercise, has been shown to enhance cognitive function through complex interactions with neural cells. This review examines how exercise-induced lactate formation acts as both an energy substrate and signaling molecule to reshape brain function, focusing on its metabolic and molecular mechanisms across different neural cell types. For that, we analyzed current literature on physical exercise-induced lactate production and its effects on brain metabolism, particularly examining lactate’s dual role in cellular energetics and signaling pathways. The review synthesizes findings from both animal and human studies investigating exercise-dependent lactate mechanisms in brain function. We conducted a comprehensive analysis of peer-reviewed literature using databases including PubMed, Web of Science, and Scopus. The search terms included combinations of “physical exercise,” “lactate,” “brain metabolism,” “cognitive function,” and “neural plasticity.” Both animal and human studies were included to provide a broad perspective on exercise-dependent lactate mechanisms in brain function. Understanding these lactate-mediated pathways is relevant for developing targeted physical exercise interventions that optimize brain health and cognitive function, potentially offering complementary therapeutic strategies for unfavorable neurological conditions.

## 1 Introduction

Physical exercise acts as a potent mediator of brain metabolism through muscle-brain communication, fundamentally transforming lactate metabolism (1). Previously, this molecule was considered just a metabolic end product and emerged as an important energy substrate and cellular signaling molecule (1–3). Through lactate dehydrogenase (LDH)-catalyzed interconversion with pyruvate, lactate establishes itself as an essential metabolic intermediate, not only meeting neuronal energy demands but also performing some regulatory functions. These functions include enhancing oligodendrocyte myelination, modulating astrocytic signaling, maintaining redox balance, and facilitating memory formation processes (2). A cell with a redox milieu can produce 10–50 times more lactate than pyruvate to shunt energy between glycolysis and mitochondria, mainly with high-intensity exercises, forming a feedback mechanism using lactate as a central molecule (3). The interest in lactate research began in the 1780s when lactate was isolated from milk sources (4). It was thought that lactate could be produced when oxygen level drops in conditions like intense exercise in the muscle or pathological situations such as stroke and cancer to show its toxic effects (5). However, in the late 1920s, this idea began to change when lactate was administered into the body, which led to an increase in glycogen deposition in the liver (6). Years later, it was revealed that the lactate could support increased respiration and metabolic responses in the brain (7). In the 1980s, it was found that lactate could support the synaptic function in the hippocampus (8). In 1994, Pellerin and Magistretti established how glutamate-mediated lactate is released in the brain through excitatory amino acid transporters 1 and 2 by proposing the Astrocyte-Neuron Lactate Shuttle Hypothesis (ANLS) (9), which explains the fundamental framework of how metabolic cooperation between astrocytes and neurons orchestrates the energy supply for neurons by explaining how glutamate uptake induces glycolysis stimulation in the astrocytes, which shuttles lactate to neurons for energy. The ANLS hypothesis suggested that lactate was a necessary fuel for highly energy-demanding neurons. However, the cellular source of lactate in the brain, the complexity of the brain’s energy metabolism, and its evolutionary strategies challenge this hypothesis. For instance, neurons exhibit metabolic duality by regulating the expression of LDHA and LDHB, allowing them to utilize either glucose or lactate as an energy source, which ensures a continuous energy supply to the neurons under various physiological conditions (10). Additionally, lactate requires one step to produce pyruvate compared to glucose, which needs ten enzymatic steps. This simplicity gives additional merits to neurons to use lactate as a primary fuel, especially in high-energy-demand conditions. However, this hypothesis failed to address the rate of lactate release at rest, the stoichiometry of glycolytic rate, and the metabolic significance during neuronal energy demands. For example, glutamate uptake-induced glycolysis in astrocytes and lactate oxidation in neurons are not metabolically significant when compared to other metabolic pathways during brain activation (11). Therefore, understanding this metabolic basis during brain activations and functions could reassure the significance of this hypothesis in brain physiology. Several studies have reported that lactate is a preferable energy source than glucose for neurons (12–16). Beyond its role in brain energy metabolism, lactate is a signaling molecule in the central nervous system (1). Although results in preclinical studies are still controversial, studies have shown that intense exercise-derived lactate crosses the blood-brain barrier (BBB) via monocarboxylate transporters (MCTs) and promotes different signaling pathways, such as Sirt1/PGC1- α/FNDC5/BDNF pathways and cAMP/PKA/CREB pathways (14, 15). Recent evidence demonstrates that the lactate-sirtuin1- peroxisome proliferator-activated receptor gamma coactivator 1-alpha (Sirt1- PGC-1α) axis induces the brain-derived neurotrophic factor (BDNF) expression, thereby enhancing synaptic plasticity and memory formation (14). However, during exercise, how exercise-induced lactate improves the metabolic functions of the brain is not well established because exercise itself requires lactate for energy compensation under high intensity. There are two possible purposes that explain this scenario in high-intensity interval training (HIIT). The first is to increase the adaptive process that can maintain brain cell homeostasis by activating lactate-mediated signaling (2). The second one is maintaining the energy status in the brain and muscle by transferring oxidized substrates from glycogenolysis (2, 9). Thus, lactate is not toxic to the brain cells, failing the old lactate paradigm theory, which defines lactate as a harmful product of anaerobic metabolism that is primarily linked with fatigue, acidosis and tissue damage, whereas it is an important competent source of glucose in the brain and maintains energy status in the muscle for exercise performance.

While current literature extensively reviews the potential role of lactate as a myokine in enhancing brain function, it often fails to address the complete array of molecular signaling pathways that may be activated by exercise. For example, Hashimoto et al. and Huang et al. discussed the potential role of lactate as a myokine (1, 13); however, they did not discuss the complete array of molecular signaling that may offer neuroprotection. For example, the interplay between lactate and specific receptors, such as HCAR1, has been well reported; however, their downstream effects in brain cells under diseased conditions remain an active area of research. To address this gap, this review examines all potential molecular signaling pathways that may be triggered by exercise-mediated lactate to enhance brain health. Additionally, the positive effects of exercise-induced lactate have been extensively studied, but the ideal dose or target blood lactate concentration for optimal brain health outcomes, as well as the factors responsible for this, are not well reported. Moreover, the contribution of exercise to increasing the bioavailability of exogenous lactate and improving brain function has not been reported in the literature. This scenario may be particularly relevant for individuals who are unable to perform high-intensity training, such as the elderly and those with compromised cardiovascular or respiratory systems. The long-term effects and safety of lactate administration remain elusive, as most studies involving lactate administration in brain disorders are acute. The role of lactate as a primary fuel source for brain cells can be influenced by manipulating various enzymes, redox molecules, and transporters. However, the specific roles of these molecules in mediating the benefits of lactate, especially during exercise, are not yet fully understood. Furthermore, there is limited reporting on drugs that target lactate-mediated mechanisms to increase lactate levels or enhance its transportation within cells in response to exercise. Additionally, the perspective of lactate as an exerkine extends beyond a muscle-centric view to a more systemic understanding, which has not been sufficiently recognized. Therefore, the purpose of this review was to address all these factors in order to suggest exercise-induced lactate as a potential modulator of brain health improvement.

## 2 Sources of lactate during exercise in the brain

Physical exercise with higher intensity increases muscle glycolysis to generate ATP, pyruvate, and nicotinamide-adenine dinucleotide (NADH). Then, pyruvate turns into lactate and is released into the blood to regenerate NAD^+^ in the mice (16). This interconversion is achieved by the LDH, which is composed of LDH-A and LDH-B (16). These subunits are varied according to the cell types of the brain. Mainly, astrocytes have LDH-A, which produces lactate from the glycogen store or glucose by glucose transporter 1 (Glut1) in the mice (17). Then, this can be released by MCT1 and MCT4 over a broad region during synaptic transmission. Oligodendrocytes can also release lactate from glycolysis by LDH-A and MCT1 to supply energy to myelinated axons (16). In addition, vigorous exercise in the mice produces muscle lactate, which can be released into the blood through MCT1 and MCT4 (15). This blood-borne lactate crosses the BBB through MCT1 to reach the brain parenchyma. Neurons have high-affinity transporters like MCT2s to bring more lactate, which can be converted into pyruvate for oxidative metabolism (17). This scenario has particular relevance during HIIT for regulating acidosis as lactate transport controls the H^+^ in glycolytic metabolism in the brain. In addition, exercise-induced lactate production from pyruvate can release one NAD^+^ molecule by using two hydrogen molecules, possibly facilitating the increase of NAD^+^ in the human peripheral blood mononuclear cells (18, 19), which could reverse the memory impairments in Alzheimer’s disease (19). Moreover, lactate can be the precursor for gluconeogenesis to restore brain glycogen after exercise (20). For example, a study reported that astrocytes possess 6-phosphofructo-2-kinase, a key enzyme for regulating gluconeogenesis (21) that can replenish glycogen storage in the brain.

## 3 Distinct roles of L- and D-lactate

Although L and D-lactate are present under healthy physiological conditions, the effects of both molecules on brain function may differ, and the mechanism of their involvement is complex to understand (22, 23). For example, L-lactate is the preferred energy source for neurons and a signaling molecule to influence several brain functions, such as learning, improving memory, synaptic plasticity, and neurogenesis. However, excess accumulation of this molecule can cause L-lactic acidosis, which disrupts normal brain functions, as evidenced by several neuropsychiatric disorders such as schizophrenia and major depressive disorder (22, 23). Meanwhile, the role of D-lactate is less acknowledged, and it can be linked to metabolic dysfunction and altering neuronal activity (24, 25). In addition, the excess accumulation of L-lactate during exercise may be inhibited by the production of D-lactate (24). For example, exercise improves the methylglyoxal metabolism in the astrocytes to induce D-lactate and GSH by the glyoxalase system (24, 25), and this scenario could inhibit the L-lactate production, but D-lactate accumulation can also interfere with the energy production by perturbing the state 3 and 4 complex of mitochondrial respiration (26). Nevertheless, D-lactate infusion impaired the memory due to its inhibitor L-lactate at MCTs, while D-lactate decreases the L-lactate uptake by the neurons and slows the L-lactate metabolism in the neurons, and the potential effects of both molecules are based on the metabolic urge of neurons (27, 28). Therefore, understanding the relationship between L-lactate and D-lactate involvement during exercise in the brain function is more complex, and establishing the distinct role of these lactate isomers could be a therapeutic target in neurodegenerative disorders.

## 4 Lactate as an exerkine in modifying brain function

Although the term “exerkine” was recently coined by Mark Tarnopolsky and colleagues in 2016 (29), evidence of these molecules’ secretion was identified several years earlier, with the discovery of lactate secretion during exercise. Since then, several exerkines have been proposed to drive exercise-mediated benefits. Lactate is one of the crucial exerkines secreted and distributed between organs or tissues by systemic circulation to improve various physiological functions. For example, exercise-induced lactate promotes the secretion of different cytokines such as TGFbeta2, which regulates brain inflammatory process and induces neuronal polarity during brain development (30). Exercise improves glycolysis, which influences the high lactate influx, causes a major redox shift in the cytoplasm of the brain cells to a reduced state, and activates redox-sensitive signaling in the brain. For example, redox changes activate the sirtuin 1, AMPK, and PGC-1α to increase mitochondrial biogenesis, improve neural development, synaptic plasticity, and axonal elongation (31). In addition, flux in the NAD^+^ to NADH ratio due to impaired glycolytic rate during exercise improves mitochondrial function and regulates inflammation and apoptosis (31, 32). Exercise-induced lactate inhibits lipolysis during exercise to regulate energy metabolism in the brain and improve neural function and neuroinflammation (32, 33). Moreover, lactate inhibits carnitine palmitoyl transferase, which dysregulates the energy homeostasis in the brain (34). However, this scenario may contribute to decreasing oxidative stress and inflammation in the brain, but it should be established whether exercise-induced lactate may inhibit the carnitine palmitoyl transferase for decreasing oxidative stress and inflammation in the brain. Exercise-induced lactate triggers the BDNF release in the brain for neurogenesis and cognitive functions. However, many questions that increase the passion of understanding exerkines, including acute and chronic exercise response in improving lactate as exerkines and viewing lactate as skeletal muscle-centric one to the systemic level, will offer new therapeutic possibilities to prevent and treat several neurological disorders.

## 5 The effects of exogenous lactate administration on brain function

Exogenous lactate administration has recently attracted attention due to its potential impact on brain function. Studies indicate that lactate from external sources can enhance brain physiology. For example, in a study by Bisri et al. (35), lactate infusion during brain injury improved cognitive function. Additionally, a study by Hwang et al. demonstrated that exercise combined with lactate infusion increased the expression of FNDC5, BDNF, and PGC1α, which enhances neuronal plasticity in the hippocampus of mice (36). Furthermore, lactate administration during exercise regulated BDNF expression via a PGC1α/FNDC5-dependent mechanism in the hippocampus (36). In rats, administration of L-lactate has been shown to improve long-term memory formation and decision-making by increasing the levels of SIRT3, KIF5B, OXR1, PYGM, and ATG7 in the hippocampus, which promotes mitochondrial biogenesis (37). Sodium lactate, when administered at high doses, may provide consistent benefits to the brain by enhancing brain metabolism and reducing intracranial pressure (38). However, individual variability, such as age, sex, and fitness level, can affect responses to lactate. It is essential to consider the optimal dosage and duration of lactate administration and the metabolic effects, long-term safety, and potential complications to understand its benefits for brain function fully.

## 6 The role of endogenous and exogenous lactate in animal models of central nervous system disorders

Lactate plays a crucial role in both endogenous and exogenous conditions within the animal models of central nervous system (CNS) disorders. For example, endogenously produced lactate by exercise in the glial cells supports the neuronal metabolism and maintains synaptic function, particularly under hypoxia conditions or increased energy demand conditions. Mainly, in ischemic events, lactate may serve as an alternative energy source to support neuron survival (39), which is in contrast to the study that reported the accumulation of lactate in the rat`s brain increased the intracerebral hemorrhages during ischemic events (40). Moreover, large-scale animal models revealed that endogenous lactate is associated with the altered brain pH to cause cognitive impairment in neuropsychiatric disorders (39). Studies have also reported that exogenous lactate administration could prevent CNS disorders. For example, as mentioned above, lactate infusion increases neuroprotection, decreases neuronal damage, and improves cognitive functions (36, 37). Exercise with lactate administration improved the BDNF regulation for increasing neuronal survival in the mice (36). Also, lactate infusion improved the long-term memory and decision-making in the rat model by increasing mitochondrial biogenesis (37). Lactate plays a vital role in the progression of AD and presents a potential therapeutic target for its treatment. For example, Zhang et al. reported that a deficit of lactate in the cerebral region impedes lactate transport from glial cells to neurons in an AD mouse model (41). Conversely, Yang et al. showed that increased glycolysis leads to elevated lactate levels, which can impair spatial cognition and accelerate the accumulation of amyloid-beta (Aβ) in the AD mouse model (42). Additionally, lactate accumulation in Parkinson’s disease (PD) may contribute to the degeneration of dopaminergic neurons (43). Interestingly, lactate exhibits both depressant and antidepressant effects in a dose-dependent manner by activating protein kinase C (PKC) and the enzymes tyrosine hydroxylase and tryptophan hydroxylase, which in turn promotes increased secretion of serotonin and dopamine in the rat brain (44, 45). Furthermore, lactate levels can aid in differentiating between epileptic and non-epileptic seizures within a specific timeframe (46). An elevated lactate level is also associated with the development and progression of spina bifida, where the neural tube fails to close completely, potentially due to lactate-induced disruption of metabolic processes in the central nervous system (CNS) (47). Additionally, lactate levels are linked to mitochondrial dysfunction and oxidative stress in conditions such as Amyotrophic Lateral Sclerosis and Multiple Sclerosis (48, 49). Overall, the dual role of lactate as both a metabolic substrate and a signaling molecule highlights its significant therapeutic potential in causing, preventing, or treating CNS disorders.

## 7 How exercise-induced lactate controls the systemic energy demand in favor of lactate

**Figure 1:**
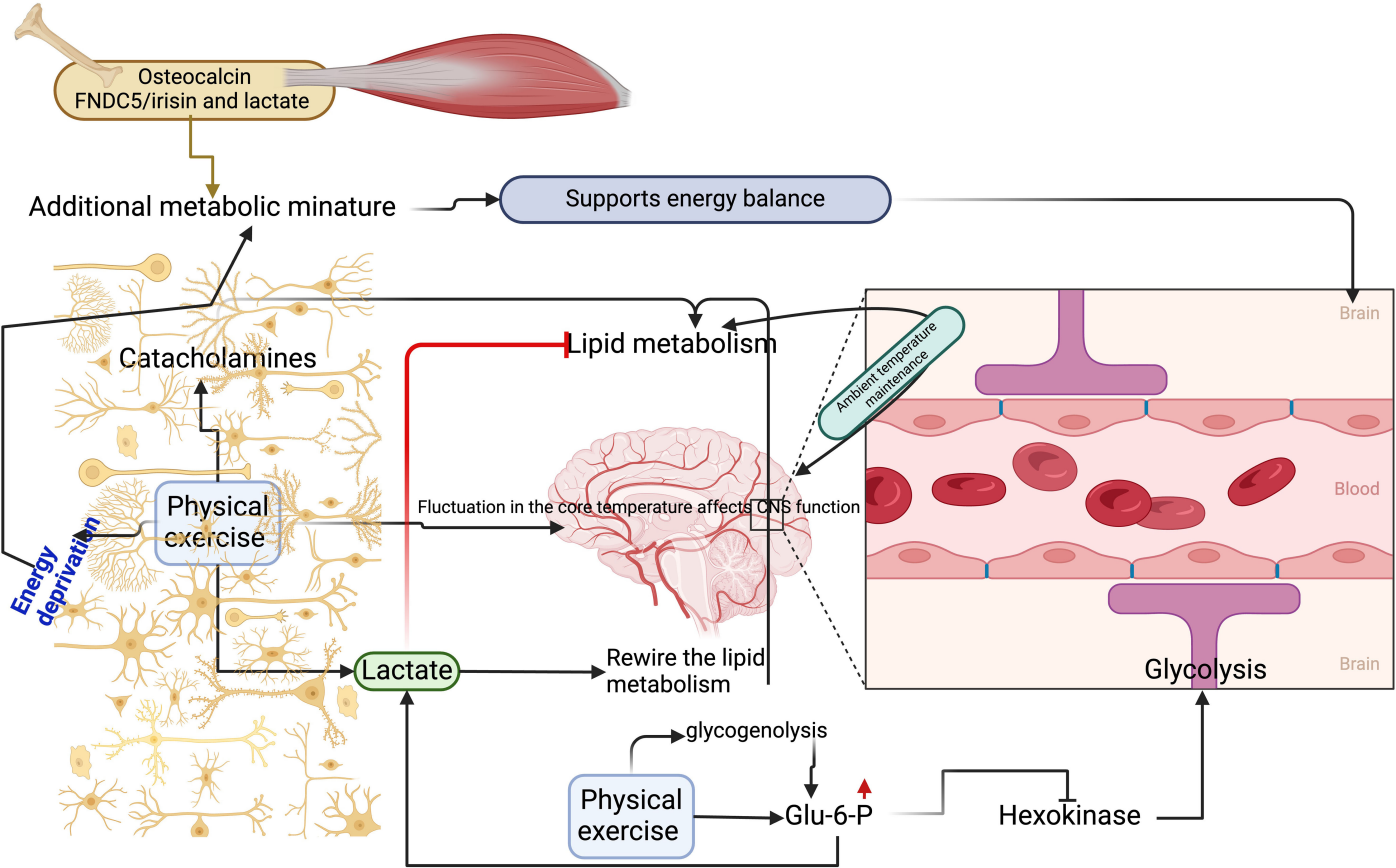
Physical exercise-induced lactate improves energy metabolism in the brain. (1) Physical exercise enhances glycogenolysis to activate Glu-6-P, which prevents hexokinase from increasing lactate concentration in the blood. (2) Lactate rewires lipid metabolism to maintain ambient temperature in the brain. (3) Physical exercise increases catecholamine levels to keep lactate within threshold levels by promoting lipid metabolism. (4) Exercise-induced blood-borne factors like osteocalcin from the bone, FNDC5/irisin, and lactate from the muscle support additional metabolic miniature to maintain energy balance during energy deprivation.

As mentioned, exercise with high intensity increases lactate formation. However, once the lactate reaches its threshold level, how it can manipulate energy demand in favor of its own way is unknown. For example, a small change in the brain temperature activates the lipid metabolism to maintain its ambient temperature. Nevertheless, an increase in lactate is linked with the inhibition of lipolysis, which might affect the brain`s ambient temperature, resulting in the functional alteration of the CNS (**Figure 1**). Exercise can also increase the brain temperature as a result of body core temperature alteration, causing fatigue induced by passive heating (50). This scenario needs further research on whether exercise-induced lactate could rewire the lipid metabolism for further release of free fatty acids for oxidation and maintain ambient temperature. In addition, initial adaptation to exercise can increase the levels of catecholamines. This can upregulate the lipolysis to maintain the lactate level within the normal range in humans, mice, and rats (5 mmol/L) (51), because the lactate threshold must be achieved to inhibit lipolysis. This feedback mechanism can be done via exercise as the lactate level can be increased up to 20 to 30 mmol/L in high-intensity exercises. In addition, the increase in lactate activates plasticity-related genes, especially in the range of 2.5 mmol/L (18, 52). However, this effect may be short-term or long-term, which needs to be explored. Moreover, it is unknown whether lactate activates this scenario in favor of its own metabolism in the brain.

## 8 Energy system disharmonizes the neural cell: role of physical exercise and lactate

**Figure 2:**
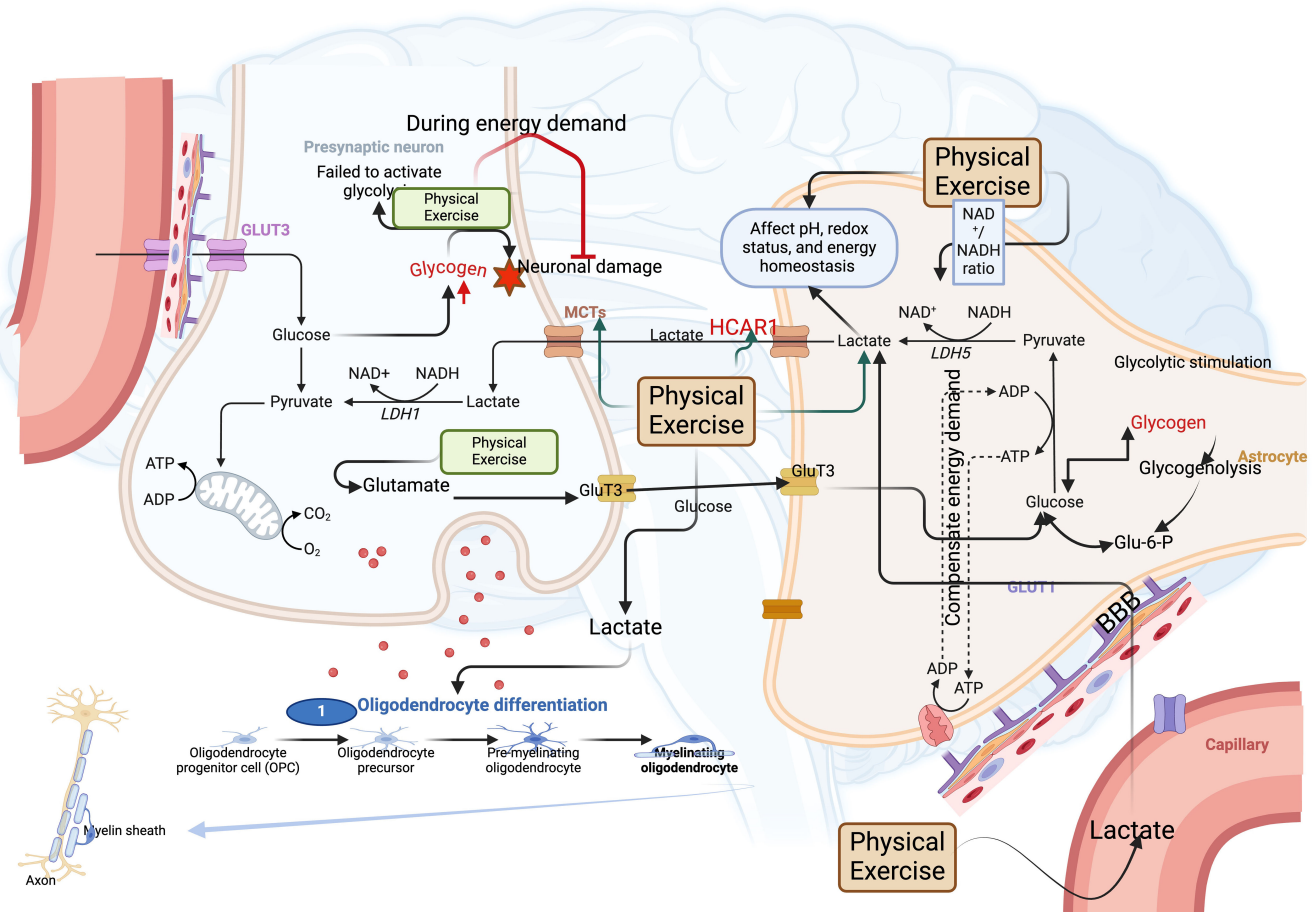
Physical exercise-induced lactate disharmonizes the energy system in the brain. (1) Physical exercise-induced lactate affects pH, redox balance, and energy homeostasis by influencing the NAD+/NADH ratio. (2) Physical exercise-induced lactate enhances HCAR1, supporting the astrocyte-neuron lactate shuttle. (3) Physical exercise-induced lactate regulates oligodendrocyte myelination. (4) Physical exercise-induced glutamate influences glycogen accumulation in neurons through lactate synthesis.

When cells harmonize the metabolic functions for energy demands, they should deal with byproducts like methylglyoxal, a key toxic intermediate of glycolysis (53). Exercise intervention modifies this byproduct by adjusting the redox balance (53, 54). For example, chronic running for 4 months improves the methylglyoxal metabolism and redox homeostasis in the cortex of the mouse brain (24), indicating the role of exercise in clearing methylglyoxal, and this effect may be time-dependent because 2 months of running exercise had no effect in improving methylglyoxal metabolism (24). However, neurons have difficulties in handling this byproduct as they do not have direct access to use these byproducts because the BBB produces more challenges when using this metabolite. Therefore, an additional metabolic miniature is required (**Figure 2**). Exercise-induced blood-borne factors like osteocalcin from the bone (55), FNDC5/irisin, lactate from the muscle, and beta-hydroxybutyrate from the liver synchronize signaling like BDNF during energy deprivation for regulating glucose balance (**Table 1**) (56). Nevertheless, before transmitting these small molecules during the energy crisis, this can cause hypoxia, as these factors need long-distance transmission (55, 57). Physical exercise can reverse this scenario by causing lactate-induced neuroprotective effects during hypoxia conditions through hypoxia-inducible factor -1 (HIF-1) activation in the mice (58). At the same time, the increase of blood lactate can be balanced by the brain cells up to 11% during exercise in humans (59, 60). In addition, other organs, such as the heart and kidneys, can either use blood glucose or albumin-bound fatty acids (57), whereas the BBB blocks the albumin in the brain. This can make brain cells rely more on glucose for ATP production than fatty acids (57). However, neurons do not possess direct access to glucose, and exercise plays a crucial role in increasing GLUT1 for the glucose diffusion process by astrocytes and endothelial cells in the rat brain (61). A single session of exercise increased the expression of Glut1 in the astrocytes and endothelial cells for energy compensation in the neurons (62, 63). Meanwhile, neurons preferentially utilize lactate during exercise, as evidenced by a 33% increase in lactate oxidation (60) and a 25% decrease in glucose uptake in humans (64). This suggests the importance of lactate as a primary fuel. This preference may be due to the absence of glucokinase, which catalyzes the first step of glycolysis and acts as a glucose sensor in the millimolar range (65, 66). In particular, this enzyme has a low binding affinity to glucose and is inhibited by glucose-6-phosphate (G6P) through feedback inhibition. The possible mechanism mediated by exercise is the breakdown of glycogen during high-intensity exercises, which could increase the accumulation of G6P to allosterically modulate the hexokinase enzyme and increase the lactate concentration in the blood (67). This can be transported across the BBB through MCTs; mainly, glial cells deliver lactate to neurons for energy homeostasis via MCT1 and MCT4 (57). Exercise affects this transportation mechanism by increasing MCTs (68). For instance, after 2 hours of acute treadmill exercise, the MCT1 increased in the cortex region, and after 5 hours of exercise, the MCT1 increased in the hippocampus and remained increased for 10 hours post-exercise in the rat brain (69). In addition, the size and the distance between the internal organelles of the neurons affect the metabolic support of neurons (70). For example, ATP turnover occurs in seconds, whereas the use of ATP between the internal structure of neurons like soma and dendrites requires about one hour as these organelles are located far from each other (57). During this scenario, lactate from the extracellular space could serve as a main energy source, which is taken up through MCT2 (57). Dendrites and axons of the neurons have greater expression of MCT2, and exercise increases the MCT2 expression. The size of the neurons can make them less capable of fulfilling their energy demand as they are cylindrical in size, possessing a larger surface area in addition to depleting their ATP pool quickly because their active state utilizes approximately sevenfold ATP (71). However, exercise is linked with increasing the size of neurons (72, 73), which might increase the ATP depletion in the active neurons, and this scenario might use other energy molecules like lactate. Nevertheless, this vicious cycle requires further understanding.

**Table 1:** Exercise-mediated signaling for lactate production and its neuroprotective role.

Signaling molecule induced by exercise	Target	Mechanism
Sirt1	Induce lacte production	Sirt1-PPGC1-alpha axis inducing the brain-derived neurotrophic factor and enhancing synaptic plasticity and memory formation.
osteocalcin	Induce lacte production	Osteocalcin target BDNF for regulating glucose balance and improve energy homeostasis.
FNDC5/irisin	Induce lacte production	FNDC5/irisin target BDNF for regulating glucose balance and improve energy homeostasis.
beta-hydroxybutyrate	Induce lacte production	Beta-hydroxybutyrate target BDNF for regulating glucose balance and improve energy homeostasis.
HIF-1 activation	Induce lactate production	Transcribes the LDH-5 activity for lactate mediated neural protection.
glucose-6-phosphate	Induce lacte production	Inhibit glucose via allosterically modulate the hexokinase to improve lactate mediated energy homeostasis.
HCAR1	Induce lacte production	Maintain the astrocyte-neuron lactate shuttle.
AMPK and adrenaline mediated by PKA	Induce lacte production	Inhibits glycogen synthase activity for lactate mediated neuroprotection.
NBCe1	Lactate release	Increase intracellular pH via facilitating carbonic anhydrase and MCTs for increasing lactate.
Glutamate signaling	Lactate production	increase the firing rate of orexin neurons by lactate mediated mechanism.

## 9 Lactate compensates glycolysis- role of physical exercise

Since neurons have limited energy reserves and require a continuous supply of glucose, they must rely on larger and more abundant cells, such as astrocytes, to utilize another energy molecule, like lactate. For example, neurons failed to activate glycolysis during energy demand, whereas astrocytes increased the glycolytic stimulation in response to greater ATP demand in the rats (74, 75). In this scenario, the lactate production and export achieve this through shunting pyruvate dehydrogenase (PDH) (76). Mainly, aerobic exercise in prolonged periods improves the lactate shuttling for energy support between neurons and astrocytes (77). This divergence occurs at the posttranslational level (74), which results in channeling the PDH by neurons into the TCA cycle, while astrocytes can phosphorylate the PDH to shunt pyruvate to lactate production (74). This can support the energy demand of the active neurons. Exercise can also activate the lactate receptor HCAR1 to maintain the astrocyte-neuron lactate shuttle (78). Another source of lactate is glycogen, which can be stored in the astrocytes. Exercising brain (exhausting treadmill exercise for 20 m/min) keeps the endurance capacity from the lactate, which is derived from the astrocytic glycogen for supporting the “selfish brain” theory (79). However, astrocytes can also require ATP synthesis in the exercising brain during brain activation; mainly, glycogenolysis can compensate for the ATP demand in the astrocytes of the rat brain (79). In another way, exercise-induced glutamate increases the Glut3 in the neurons for enhancing glucose uptake and glycogen synthesis in the astrocytes, suggesting the involvement of glutamate in maintaining ATP synthesis during exercise, mainly in the cortex area of the rat brain (79). Neurons in a healthy state do not store glycogen. Nevertheless, they have glycogen synthase in the inactive state, and mutation in the glycogen synthase leads to aberrant accumulation of glycogen in the neurons and causes Lafora disease, characterized by ataxia and dementia (57). Moreover, exercise could possibly reverse this condition by creating a temporary energy demand in the active neurons. Exercise inhibits glycogen synthase activity by activating AMPK and adrenaline mediated by PKA (80). Additional complexity comes from the mismanagement of the lactate shuttle in the oligodendrocytes, causing axonal damage because it can utilize more lactate for lipid synthesis (81, 82). Aerobic exercise for six months decreases the abnormal myelination and abnormal differentiation of oligodendrocytes in the mice (83), possibly through improving lactate metabolism by displacing glucose, showing the adaptive value of exercise that allows the brain to perform higher even with an individual with hypoglycemic condition (79, 84). In addition, the volume of the astrocytes surrounding the neurons has several advantages regarding lactate concentration, which can be readily available to displace glucose as fuel in hypoglycemia conditions. However, the lactate clearance from the glycolysis is regulated by the ATP/ADP ratio, which is determined by the physical exercise-induced mitochondrial activity and further energy consumption that can decrease the pyruvate and lactate levels.

## 10 Exercise regulates the lactate transients as an intracellular messenger

Exercise has been linked with supporting lactate-mediated signaling by balancing lactate clearance via triggering various molecular events in the brain. The transient activity of lactate is around 1–5 mins, and within this time, lactate should carry out all its molecular signaling activities (57). High-intensity exercise can increase the lactate transient activity, in particular, acute resistance exercise increases the lactate transients in the postprandial period in humans (85), possibly inducing neural stimulation during exercise parsimoniously extending this lactate transient using lactate, glucose, and oxygen (84, 86). Nevertheless, the lactate transient speed and its diffusion range in the neuron and around the neurons may be due to the increase of lactate production by exercise intensities (86, 87). Importantly, the increase of lactate must be cleared either via BBB or oxidative metabolism (88), and exercise could increase oxygen consumption for successful clearance. As mentioned, exercise stimulates glutamate release, which can increase glucose use and lactate generation through Na^+^/glutamate co-transporter (89). As soon as the stimulation of the glutamate occurs, Glut1 is activated through Na^+^/glutamate co-transporter with the help of intracellular Ca^2+^ and Na^+^ (90–92). However, a higher level of glutamate buildup is toxic to the brain, and exercise could reduce the buildup of glutamate, showing the double-faceted role of exercise (93). Moreover, exercise-induced extracellular K+ improves the glycolytic rate by altering electrochemical forces by increasing NBCe1 (94, 95). This can increase the intracellular pH by facilitating carbonic anhydrase and MCTs to increase glycolysis and lactate release in humans and animal models (96, 97), suggesting that exercise-induced activation of these molecules increases lactate transient activity. However, the persistence of this lactate transient ascending phase after exercise may be reversed immediately, requiring further research.

## 11 The specific role of lactate in brain functions: effect of exercise

Lactate affects the brain in every possible way by modifying redox balance, influencing local metabolism, and maintaining intracellular pH. This has started from the cellular level phenomena to the organ levels, including regulating the oligodendrocyte myelination by lactate (98), lactate-mediated Ca2+ signaling of astrocytes (90), and lactate-mediated organ-level process to memory formation (99). However, the link between the molecular signals and their effect on the organ level is undetermined. Physical exercise is one of the ways to organize these molecular signaling events via elevating lactate to produce organ-level effects (5). This can improve the lactate uptake through MCTs (68), and this scenario carries more protons into the cell, causing acidification within the cell (100), and potentially influencing the ion channels and receptors (5, 57). In addition, physical exercise-induced lactate increases the ratio of NADH/NAD^+^ when it is converted into pyruvate by LDH (101), and this scenario virtually affects the pH, redox status, and energy homeostasis of the cell (57). For example, regular physical exercise activates the KATP channel upregulation in the rats (102), which can possibly coordinate the coupling of astrocyte–orexin neurons for offering neuroprotection (103), suggesting the exercise-induced lactate may be a paracrine factor for signaling activity of the brain (104). Mainly, exercise-induced fuels like lactate can compensate for the energy substrate supply in the mouse brain (36) by stimulating astrocytic glucose metabolism and glutamate signaling to increase the firing rate of orexin neurons in the mice (105, 106), while the activity of orexin is necessary to maintain the exercise performance (106). This can increase the hepatic glucose uptake through the sympathetic nervous system (103), and this can match up the energy requirement during exercise in a positive feedback mechanism. However, brain lactate has been obtained from several cellular wastes to use as fuel that has come from a longer way to the brain via manipulating many enzymes, redox molecules, and transporters, and addressing these molecules will reveal more benefits of lactate and how physical exercise coordinates this.

## 12 Is exercise-mediated lactate a therapeutic target for brain diseases?

Exercise-mediated lactate accumulation for regulating metabolic processes could establish a framework for suggesting exercise as a main intervention therapy in brain research. For example, Ischemic injury interrupts oxygen delivery and causes hypoxia, which can induce lactate-mediated neuroprotection. To induce lactate accumulation, triggering LDH-A activity and disrupting LDH-B can improve the LDH-5 stimulation, which can convert pyruvate to lactate reversibly (107). This can be mainly mediated by physical exercise-induced HIF-1 that transcribes the LDH-5 activity. So, targeting exercise-mediated HIF-1 could offer lactate-mediated neuroprotection by improving LDH-A activity (107). Next, blocking NAD^+^ could slow down the glycolysis that can increase neurodegenerative diseases, such as ataxia telangiectasia and Cockayne syndrome. Designing a strategy to augment NAD^+^ could prevent these conditions via a lactate-mediated mechanism. Exercise-triggered NAD^+^ precursors such as sirt1 could increase NAD^+^ augmentation that offers lactate-mediated neuronal survival and improves cognitive function (108, 109). In addition, drugs that can induce lactate production and transportation within the cells could effectively prevent brain-related diseases. However, no studies were found in the literature about using these drugs with exercise on lactate production. Therefore, addressing the careful use of these drugs along with exercise could provide lactate-mediated neuroprotection.

## 13 Conclusion

Physical exercise-induced lactate may improve brain cells in several ways, including maintaining redox balance via improving LDH activities, enhancing local metabolism, and regulating acidosis. This can provide lactate-mediated neuroprotection. In addition, lactate itself becomes an energy source for improving exercise performance, mainly in higher-intensity conditions. However, these functions are mainly achieved via lactate as a signaling molecule or as a metabolic substrate to compensate for energy demand in the muscle and brain requires further research. Moreover, lactate has been reported as a therapeutic target in many clinical conditions and triggers a specific drug response similar to glucose. Therefore, exploring lactate as a signal transducer could facilitate this molecule as a valuable target for treating or being a prognosis marker for several brain diseases, including many neurodegenerative conditions.
